# Oligosaccharide equine feed supplement, Immulix, has minor impact on vaccine responses in mice

**DOI:** 10.1038/s41598-021-04132-8

**Published:** 2022-01-12

**Authors:** Ida Wang Henriksen, Josue Leonardo Castro Mejia, Caroline Martha Junker Mentzel, Frederikke Lindenberg, Axel Kornerup Hansen

**Affiliations:** 1 Department of Veterinary and Animal Sciences, University of Copenhagen, Ridebanevej 9, 1870 Frederiksberg C, Denmark; 2 Brogaarden Aps, Lynge, Denmark; 3 Department of Food Science, University of Copenhagen, Rolighedsvej 26, 1958 Frederiksberg C, Denmark

**Keywords:** Immunology, Microbiology

## Abstract

Several mammalian species are vaccinated in early life, but little is known about the effect of diet on vaccine response. Oligosaccharides are increasingly proposed as dietary supplement for young individuals due to their anti-inflammatory potential elicited through modulation of gut microbiota (GM). Also, diet, e.g. the size of the fat fraction, is known to modulate the GM. We tested if an oligosaccharide diet (Immulix) and/or increased dietary fat content affected antibody titers to a tetanus vaccine in 48 BALB/cJTac mice through GM modulation. Female mice had significantly higher IgG titers with higher variation compared to male mice. The effects of Immulix and/or increased fat content were minor. Immulix negatively affected IgG titers in male mice four weeks after secondary vaccination but upregulated *Il1b* gene expression in the spleen. Immulix had a downregulating effect on expression of *Cd4* and *Foxp3* in ileum only if the mice were fed the diet with increased fat. The diet with increased dietary fat increased *Il1b* but decreased *Cd8a* gene expression in the spleen. Immulix and diet affected GM composition significantly. Increased dietary fat content upregulated *Lactobacillus animalis* but downregulated an unclassified *Prevotella* spp. Immulix decreased Lactobacillales, *Streptococcaceae* and *Prevotellaceae* but increased *Bacteroides.* It is concluded that in spite of some minor influences on immune cell markers, cytokines and IgG titers Immulix feeding or increased dietary fat content did not have any biologically relevant effects on tetanus vaccine responses in this experiment in mice.

## Introduction

Diet exerts modulatory effects on gut microbiota (GM) composition in several mammalian species^1^. In healthy humans, diet changes, on terms as short as five days, the GM composition with animal-based diets having greater impacts on GM composition than plant-based diets^2^. Prebiotics, which were initially defined as: *“…a non-digestible food ingredient that beneficially affects the host by selectively stimulating the growth and/or activity of one or a limited number of bacteria in the colon, and thus improves host health”*^3^, have proven to support GM recovery after dysbiosis caused by antibiotics^4^, infections^5,6^, ageing^7,8^ or surgery^9^. Several studies have reported increased relative abundances of *Bifidobacterium* and *Lactobacillus*^10,11^ as well as *Akkermansia*^6,12^ in the GM of animals fed oligosaccharides, which are among the most frequently used and researched prebiotics. These bacteria counteract dysbiosis, have various positive effects on the immune system, such as increased production of antibodies^10^, cytokines^13–15^, regulatory T (T_reg_) cells^16^ and short chain fatty acids (SCFAs)^17^, and they have been correlated to reduced risk of autoimmune diseases such as colitis, diabetes and arthritis^18–21^ as well as asthma and allergies^22,23^. Examples of their use are milk formulas, such as NAN Sensilac 1 from Nestlé (Vevey, Switzerland) for human babies with galacto- and fructo-oligosaccharides added^24^. Another example is the prebiotic feed supplement Immulix, which was developed for mares and foals and consists of a mixture of mannan- and fructo-oligosaccharides, inulin and some anti-caking and carrier substances^12^. Immulix was launched as a result of thorough research of the equine GM^12,16,25^ and has been related to increased abundances of *Akkermansia* spp. and *Campylobacter* spp. in the equine gut^12^. In horses fed this diet, Clostridiales spp. and *Akkermansia* spp. have been positively related to regulatory immunity^16^, through upregulation of genes coding for the anti-inflammatory cytokines IL-10 and TGF-β and the T_reg_ cell transcription factor FOXP_3_ as well as downregulation of genes coding for the proinflammatory cytokine IL-12 in ileum and mesenteric lymph nodes (MLN)^16^.

Vaccination is an indispensable tool for prevention of infectious diseases, and varying or even lack of efficiency poses problems in the control of global diseases in both human and domestic animals^26,27^. Since Immulix is a product for foals, which are routinely vaccinated against tetanus, influenza and herpesvirus at the age of five to six months^28^, it would be interesting to test how Immulix feeding affects the vaccine response in horses, by using a murine model. Knowledge about effects, as well as possible side-effects, of a product increases its safety in use. Besides that, increased knowledge on the effects of oligosaccharides on GM and the immune system, can be useful for other mammals, including humans, and contribute to further research within this field. Oligosaccharides are known to stimulate production of T_reg_ cells and IL-10 in horses. T_reg_ and IL-10 are among the most important components of immunoregulatory mechanisms aimed to control inflammation i.e. primarily exert anti-inflammatory impact, why it is reasonable to hypothesize that it may have a dampening impact on vaccine response. Oppositely, due to specific positive influences on B cells, IL-10 might exert a positive impact on vaccine responses^29^. A meta-analysis by Lei, et al.^30^ concluded that the intake of pre- and/or probiotics, enhance serum immunoglobulin (Ig) titres to influenza vaccines in adult humans, indicating increased immune responsiveness ^31^. Similar results have been reported in piglets and broilers^32,33^. A study by van den Elsen, et al.^34^ demonstrated that early oligosaccharide administration (either starting from fertilization through the mother, from birth or from weaning), enhanced IgG titers to influenza vaccines in male but not female mice compared to controls. Even though exact mechanisms appear unclear, GM seems to play a critical role due to the fact that oligosaccharides evidently correlate with specific beneficial bacteria that correlate with vaccine responses. Zhang, et al.^10^, reported increased *Bifidobacterium*, *Lactobacillus* and *Bacteroides* abundances in the GM of oligosaccharide fed mice, which correlated positively with serum IgG concentrations.

Basic dietary macronutrient levels may also influence GM, and eventually thereby vaccine responses. Moreno-Indias, et al.^35^ showed that the dietary fat content (approx. two times more compared to a standard rodent chow diet) significantly altered GM composition of mice and enhanced the percentage of B cells in Peyer’s patches (PP’s) and *Cd8a* expression in ileum^35^. Oil supplements in horse diets have furthermore proven to affect immune functions in horses^36^. It, therefore, seems relevant to be aware that responses to both an oligosaccharide supplement and a vaccine may differ according to the diet fed and that especially the fat proportion of the diet may have an influence on the immunological outcome through GM manipulation.

The aim of this study was, therefore, to investigate if Immulix in mice fed diets with standard or increased fat content would influence the immune responses to a tetanus vaccine in a murine model and to assess how the GM were affected for a better understanding of the interplay between GM and the immune system. We used antibody response to the tetanus vaccine as primary read-out. Based on previous results from the literature, the effect of oligosaccharides could be both up- and down-regulatory on the immune system and therefore, a two-sided hypothesis was tested. We tested our hypothesis in mice fed either a diet with an ordinary rodent macronutrient profile (MP = murine profile) or a diet with a more human macronutrient profile (HP = Human profile), i.e. with increased amount of fat.

## Materials and methods

This study was approved by the Animal Experiments Inspectorate, Ministry of Food, Fisheries and Agriculture, Denmark. All housing, maintenance and experimental procedures were carried out according to the EU directive 2010/63/EU on the protection of animals used for scientific purposes and The Danish Animal Experimentation Act (LBK 474 from 15/05/2014). This study was reported in accordance with the ARRIVE guidelines^37^.

### Study design and sampling

**Figure 1:**
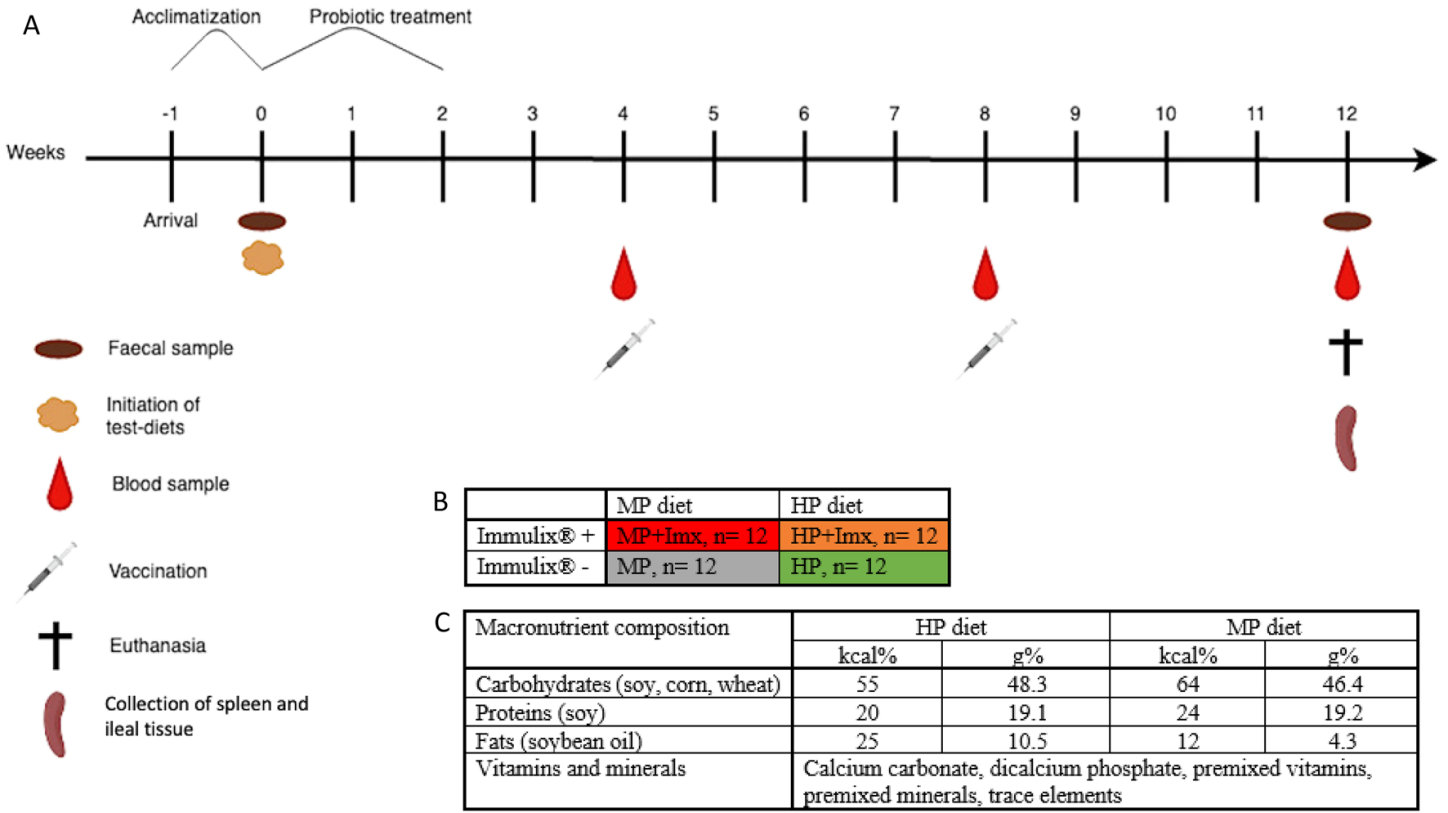
(**A**) Timeline indicating major experimental procedures. (**B**) Feeding groups were denominated as MP + Imx; HP; MP; and HP + Imx, respectively. (**C**) Macronutrient composition of the two diets used. Created with Biorender.com.

24 male- and 24 female BALB/cjTac mice (Taconic Europe, Lille Skensved, Denmark), aged three to four weeks at arrival, were randomly allocated in four feeding groups designated MP (mouse profile); HP (human profile); MP + Imx; HP + Imx (Fig. 1B). Each group contained six males and six females co-housed according to sex in polycarbonate 1290D Eurostandard type III cages supplied with Tapvei aspen bedding, Tapvei wooden chewing block, disposable Smart Home shelters, Mini Fun Tunnels, Nestlet nesting material and Enviro-dri (Brogaarden, Lynge, Denmark) at temperatures of 22 ± 2 ◦C, humidity of 55% ± 10%, air changing 15–20 times/hour and a 12 h light cycle. The sample size was founded on a power analysis, that revealed a sample size of 12 with 90% power and 5% significance level, assuming effect size of 28 U/ml and standard deviation (SD) of 20 U/ml based on comparable results from a previous study of the same type^38^, and a two-sided-test. Taking this into account, in combination with experience from previous comparable studies and practical considerations, a sample size of 12 mice per group was deemed appropriate.

During the 12 weeks experimental period (Fig. 1A), the MP and MP + Imx groups were fed a standard Altromin 1324 diet with a macronutrient composition as normally recommended for mice, i.e. a fat content of 4.3 g % (here designated “MP diet” (mouse profile); Brogaarden, Lynge, Denmark), while the HP and HP + Imx groups were fed a modified Altromin 1320 diet with a macronutrient profile as normally recommended for humans, i.e. a fat content of 10.5%^35^ (here designated “HP diet” (human profile); Brogaarden, Lynge, Denmark). In the HP diet, the macronutrient composition was changed to match a diet recommended for humans, similar to the “human profile (HP)” diet previously applied^35^. Especially the fat proportion was increased (from 4.3% g to 10.5% g) in the HP diet (Fig. 1C). The MP + Imx and HP + Imx groups were additionally fed Immulix (6.3 g per 100 g feed; Brogaarden, Lynge, Denmark). During the first 14 days of the experiment, the mice received probiotics (a mix of *Bifidobacteria* and *Lactobacilli*; Udo’s Choice, Super Infant 3–5 years; FMD, Burnaby, Canada) through drinking water (0.1 g/100 ml water) in order to help the establishment of *Bifidobacteria* colonies in the mice, which laboratory mice often lack^39^ (Fig. 1A). The mice were weighed every second week and vaccinated subcutaneously in the lumbar region (0.3 Lf/mouse/vaccination) at week 4 and 8 with an equine tetanus vaccine (EQUIP T. VET; Orion Pharma Animal Health, Espoo, Finland). Faeces were sampled in sterile 1.5 ml Biosphere SafeSeal tubes by voluntary defecation at week 0 and 12. The samples were temporarily stored on ice, and thereafter at − 80 °C until analysis of microbial DNA. Blood was sampled at week 4 and 8 by puncturing the submandibular vein and at week 12 by puncturing the retro-orbital sinus. Blood samples were stored at room temperature for approx. one hour to allow coagulation and thereafter at 4 °C until centrifugation (5000 X g, 10 min, 5 °C). Serum was transferred to individual 1.5 ml Biosphere SafeSeal tubes and stored at − 20 °C until analysis of IgG by ELISA. The mice were euthanized at week 12 by cervical dislocation. Immediately hereafter, the spleen and 1 cm of the distal ileum were collected and placed in individual sterile 1.5 ml Biosphere SafeSeal tubes with RNAlater and temporarily stored on ice, before being stored at − 80 °C until analysis of gene expression by qPCR.

### Serum IgG by ELISA

Anti-tetanus-specific serum IgG titres were assessed using a pre-coated ELISA kit (Mouse Anti-Tetanus Toxin/Toxoid IgG ELISA kit 930–130-TMG; Alpha Diagnostic International, San Antonio, TX, USA) by following the manufacturer’s protocol. Serum were diluted in order to obtain IgG concentrations by interpolation from a calibrator curve. Samples from week 4 were tested in singles, while all other samples, standards and controls, were tested in duplicates. OD was measured on an Epoch Microplate Spectrophotometer at 450 nm and analysed with Gen5™.

### Tissue gene expression by qPCR

Spleen and ileal tissue samples were homogenized on a FastPrep with 6.5 m/s for 45 s (MP Biomedical, USA) in the manufacturer’s tubes using 0.6 mg acid-washed glass beads (1001982996; Sigma Life Science, Missouri, USA) in the lysis buffer included in the MagMAX™-96 total RNA Isolation Kit (AM1830; Thermo Fisher Scientific, Waltham, MA, USA). Homogenates were stored at − 80 °C until RNA purification on a MagMAX™ Express Magnetic Particle Processor, using MagMAX™-96 total RNA Isolation Kit, following manufacturer’s instructions. RNA concentration was assessed with a NanoDrop 1000 Spectrophotometer and RNA integrity was evaluated on a 1.4% agarose gel. cDNA was synthesized from ∼500 ng RNA with a High-Capacity cDNA Reverse Transcriptase kit (Thermo Fisher Scientific, Waltham, MA, USA), following manufacturer’s instructions. cDNA was amplified and measured in duplicates with a C1000 Touch™ Thermal cycler and CFX96™ Real-Time PCR Detection System using the following TaqMan gene expression assays specific for the candidate genes: *Il6 (Mm00446190_m1); Il1b (Mm00434228_m1); Il10 (Mm00439616_m1); Tnf*α *(Mm00443258_m1); Foxp3 (Mm00475162_m1); Cd4 (Mm00442754_m1); Cd8a (Mm01182107_g1); Cd19 (Mm00515420_m1)* as well as selected reference genes for spleen: *Actb (Mm00607939_s1); Gadph (Mm99999915_g1)* and ileum: *Actb (Mm00607939_s1); Sdha (Mm01352366_m1)* and TaqMan Fast universal PCR Mastermix (Thermo Fisher Scientific, Waltham, MA, USA). For each investigated gene, a non-template control and two no-reverse transcriptase controls (-RT) were included. Cycle of quantification (Cq) values were obtained with CFX Maestro Software (Bio-Rad, Hercules, CA, USA). Gene assays binding to gDNA in the -RT control was excluded from further analysis (*Gapdh* in spleen). GenEx 6 (MultiD Analyses AB, Gothenburg, Sweden) was used for qPCR data transformation. Cq values were normalized to the reference genes *Actb* (spleen) and *Sdha* + *Actb* (ileum), which was appraised suitable for normalization using the built-in functions geNorm^40^ and NormFinder^41^. For each gene, normalized expression levels were set relative to the sample with lowest expression to establish relative quantities (RQ) and were log2 transformed before statistical testing.

### Gut microbiota characterization by 16S rRNA sequencing

Faecal DNA was isolated using Bead-Beat Micro AX Gravity kits (A&A Biotechnology, Gdynia, Poland) following manufacturer’s protocol. DNA quality and concentration were assessed with a Varioskan Flash machine using an Invitrogen Qubit™ 1X dsDNA HS Assay Kit (Thermo Fisher Scientific, Waltham, MA, USA). Amplification of the V3 region of the 16S rRNA gene was performed on a SureCycler 8800 Thermal Cycler using the following primers: nxt338F and nxt518R. The PCR mixture containing 5 μl PCRBIO HiFi buffer 0.5 µl primer mix (5 µM each); 0.25 μl PCRBIO HiFi Polymerase (2 u/μl); 1 μl bovine serum albumin (1 ng/μl) ; 1 μl formamide; 12.25 μl nuclease-free water; and 5 μl of extracted DNA (1–2 ng/μl) giving a total volume of 25 µl, was treated as follows: 95 °C for 2 min; 33 cycles of 95 °C for 15 s, 55 °C for 15 s and 72 °C for 20 s; 72 °C for 4 min. DNA was validated by electrophoresis on a 1.5% agarose gel for 45 min at 120 V. Adaptors and indices were incorporated to the amplicon by treating a mixture containing 5 μl PCRBIO HiFi buffer; 0.25 μl PCRBIO HiFi Polymerase (2 u/μl); 13.75 μl nuclease-free water; 4 µl of a unique combination of Illumina Index primers (P5 and P7, Nextera XT DNA Library Preparation Kit; Illumina, San Diego, CA, USA); and 2 µl PCR product, giving a total volume of 25 µl as follows: 95 °C for 1 min; 13 cycles of 95 °C for 15 s, 55 °C for 15 s and 72 °C for 15 s; 72 °C for 5 min. Amplicons with adaptors and indices were purified with a Biomek 4000 automated laboratory workstation by using AMPure XP beads and quantified with a Varioskan Flash machine by using an Invitrogen Qubit™ 1X dsDNA HS Assay Kit (Thermo Fisher Scientific, Waltham, MA, USA). Finally, 5 μl of all amplicons with adaptors and indices were pooled in one Eppendorf tube followed by sequencing on an Illumina NextSeq 550 platform (Illumina, San Diego, CA, USA).

Raw data, containing pair-ended reads with corresponding quality scores, were initially merged and trimmed with the settings: -fastq_minovlen 100, -fastq_maxee 2.0, -fastq_truncal 4, -fastq_minlen 130. Quantitative Insight Into Microbial Ecology (QIIME; v 1.9) open source software package^42^ was used for identifying unique reads and deconvoluting from chimeric reads as well as construction of *de-novo zero-radius* OTUs by utilizing the UNOISE pipeline^43^. The EzTaxon-e procaryotic 16S rRNA gene sequence database was used as a reference^44^.

### Statistical methods

All data were initially tested for normal distribution (Anderson–Darling test) within feeding groups and equal variances (Levene’s test). If criteria for parametric testing were not fulfilled, data were ranked before further statistical testing. Statistics were unless otherwise mentioned performed with Minitab Statistical Software version 19 (Minitab, Coventry, UK).

Weight data (AUC), IgG, Log2(RQ) and OTUs were analysed by a multifactorial general linear model ANOVA on factors Immulix, diet and sex including interactions. Interactions were compared with Fisher’s least significant difference test. Whether Immulix feeding resulted in significantly more or less high responders (cut-off defined as median + 2 × SD) in IgG titers was tested with Fisher’s exact test. Relative abundance at each taxonomic level was based on rarefied OTU-tables and was only included for statistics if at least one of the groups mean (+ Immulix vs. -Immulix; MP vs. HP) was > 1%. One OTU was analysed with Kruskal–Wallis test with Dunn’s post hoc test due to unequal variances of ranked data (Graphpad Prism8; GraphPad Software, San Diego, CA, USA). Each set of p-values (Immulix or diet) was corrected for False Discovery Rate (FDR) (Two-stage linear step-up procedure of Benjamini, Krieger and Yekutieli, desired FDR = 5%) generating “q-values” based on the newly defined statistical significance level: p < 0.003 (Graphpad Prism 8; GraphPad Software, San Diego, CA, USA). Bray–Curtis dissimilarities (beta-diversity) were calculated from rarefied OTU-tables (10,000 sequences/sample) and visualized with three-dimensional principal coordinate analysis (PcoA) plots. Differences in beta-diversity (analysis of similarities), and alpha-diversity (t-test) were calculated using functions implemented in QIIME; v 1.9 toolbox^42^.

It was additionally tested if correlations existed between OTUs and remaining data. p-values were obtained with Pairwise Pearson Correlations and were subsequently FDR-corrected as described above.

## Results

### Female mice had significantly higher and more varied IgG titers compared to male mice and Immulix significantly decreased IgG titers in male mice

**Figure 2:**
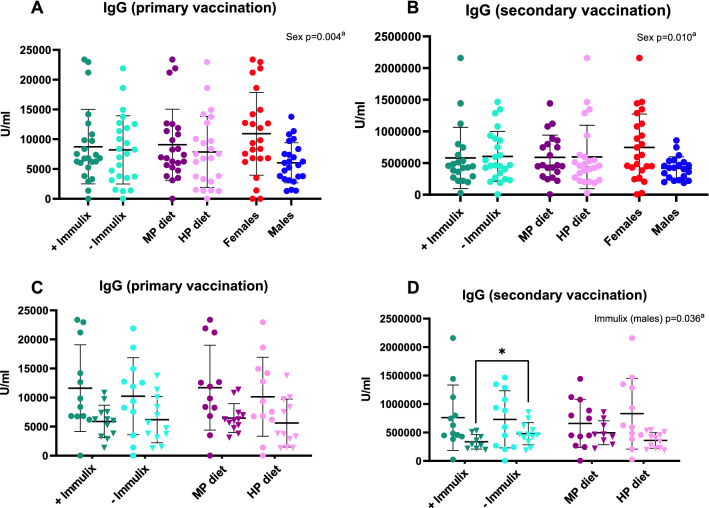
Effect of Immulix, diet and sex on IgG titers four weeks after primary (**A** and **C** ) and secondary (**B** and **D**) vaccination. Plots show individual values and group mean with standard deviation. ^a^*p*-values of multifactorial general linear model ANOVA on factors Immulix, diet and sex either without (A and B) or with (C and D) division according to sex within the factorial groups.

Samples from week 4 were all negative in tetanus specific IgG, as expected. Immulix or diet had no significant effects on IgG titers four weeks after primary or secondary vaccination (Fig. 2A and B). However, four weeks after vaccination females demonstrated significantly higher (primary, p = 0.004; secondary, *p* = 0.010) and more varied (*p* = 0.005) tetanus-specific IgG titers compared to males (Fig. 2A and B). IgG titers four weeks after secondary vaccination were significantly lower for Immulix fed male mice, compared to males not fed Immulix (p = 0.036; Fig. 2D). This effect was not seen 4 weeks after primary vaccine (Fig. 2C). Immulix feeding did not result in more or less high-responders (cut-off defined as mean + 2 × SD) four weeks after primary (*p* = 0.234) or secondary (*p* > 0.999) vaccination.

### Immulix feeding increased *il1b* in spleen and downregulated gene expression in mice on a human profile diet

**Figure 3:**
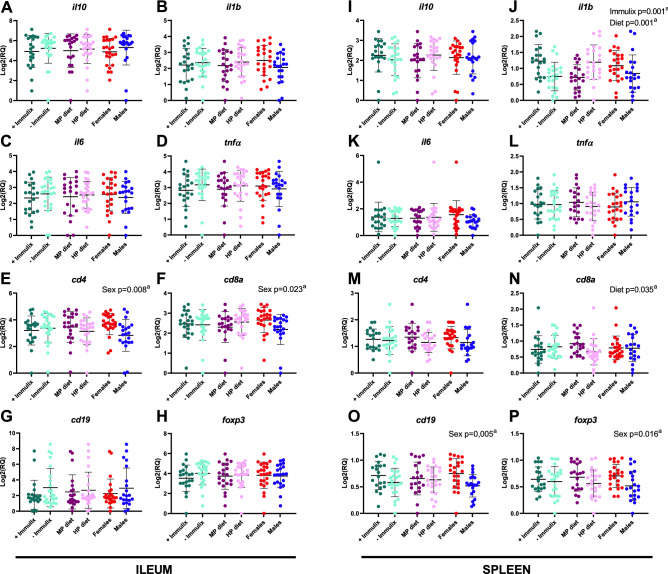
Significant effects of sex, diet and Immulix on gene expression in ileum (A-H) and spleen (I-P), expressed as log2 transformed relative quantities (RQ). Bars indicate group mean with standard deviation. ^a^ p-values obtained by multifactorial general linear model ANOVA on factors Immulix, diet and sex.

**Figure 4:**
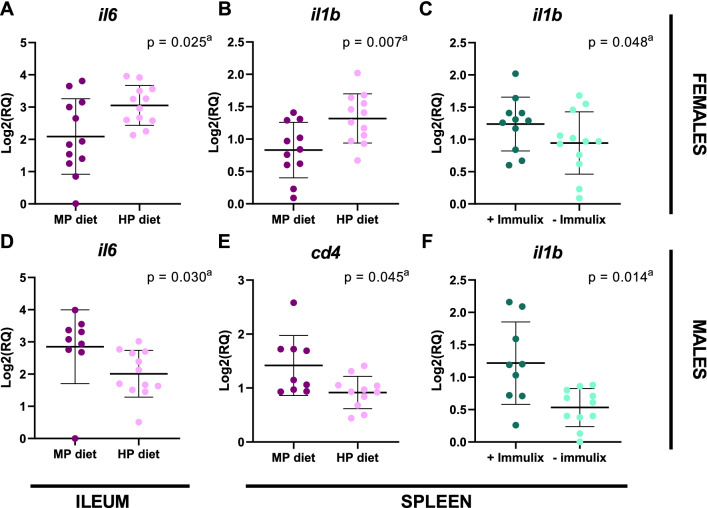
Significant effects of diet (**A**, **B**, **D** and **E**) and Immulix (**C** and **F**), within females (**A**–**C**) and males (**D**–**F**), on gene expression in ileum (**A** and **D**) and spleen (**B**, **C**, **E** and **F**), expressed as log2 transformed relative quantities (RQ). Bars indicate group mean with standard deviation. ^a^*p*-values obtained by multifactorial general linear model ANOVA on factors Immulix and diet.

**Figure 5:**
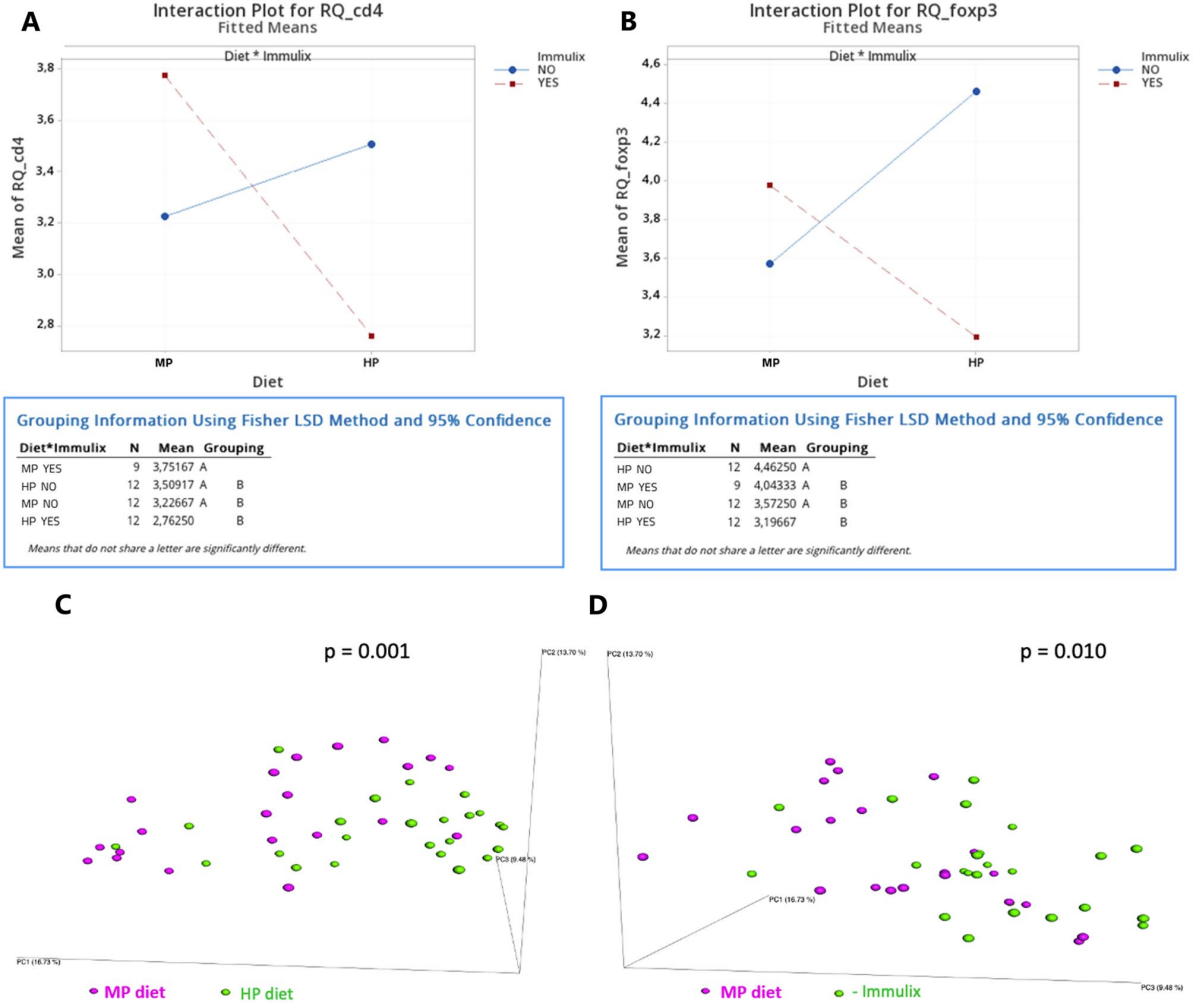
Interaction plots (multifactorial general linear model ANOVA) between Immulix and diet for cd4 (**A**) and foxp3 (**B**) log2 transformed relative quantities (RQ) in ileum. Both plots show that on the HP diet, Immulix had a downregulating effect on the gene expression. PcoA plots based on Bray–Curtis dissimilarities of faecal gut microbiota from week 12. (**C)**: Visualizing MP and HP diet clusters. (**D**): Visualizing + Immulix and -Immulix clusters. *p*-values obtained by analysis of similarities.

Females demonstrated significantly higher expression of *Cd4* (p = 0.008; Fig. 3E) and *Cd8a* (p = 0.023; Fig. 3F) in ileum and *Cd19* (p = 0.005; Fig. 3O) and *Foxp3* (p = 0.016; Fig. 3P) in spleen compared to males. Feeding with HP diet, instead of MP diet, resulted in significantly higher expression of *Il1b* (p = 0.001; Fig. 3J) but lower expression of *Cd8a* in spleen (p = 0.035; Fig. 3N). Finally, Immulix feeding resulted in higher expression of *Il1b* in spleen (p = 0.001; Fig. 3J), compared to no Immulix feeding. Neither Immulix, diet or sex had any effects on expression of *Il10* (Fig. 3A), *Il1b* (Fig. 3B), *Il6* (Fig. 3C), *Tnf*α (Fig. 3D), *Cd19* (Fig. 3G) and *Foxp3* (Fig. 3H) in ileum or *Il10* (Fig. 3I), *Il6* (Fig. 3K), *Tnf*α (Fig. 3L) and *Cd4* (Fig. 3M) in spleen. Because sex was found to have significant effects on expression of some genes, the effect of Immulix and diet was tested within each sex. Feeding the HP diet increased *Il6* (p = 0.025; Fig. 4A) and *Il1b* (*p* = 0.007; Fig. 4B) expression in females but decreased *Il6* (*p* = 0.030; Fig. 4D) and *Cd4* (p = 0.045; Fig. 4E) expression in males, compared to the MP diet. Furthermore, Immulix feeding upregulated *Il1b* expression in both females (*p* = 0.048; Fig. 4C) and males (*p* = 0.014; Fig. 4F). There were significant interactions between Immulix and diet for *Cd4* (*p* = 0.046) and *Foxp3* (*p* = 0.027) in ileum (Fig. 5A and B). Means compared with Fisher’s least significant difference test deposed that Immulix, in combination with the HP diet, had a downregulating effect on expression of *Cd4* (Fig. 5A) and *Foxp3* (Fig. 5B) in ileum.

### Diet and Immulix altered gut microbiota composition

**Figure 6:**
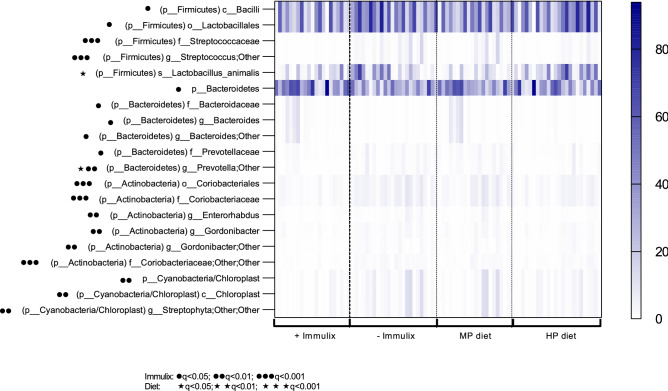
Heatmap presenting taxa affected significantly (q < 0.05) or tended to be affected (*p* < 0.05) by either diet or Immulix. p-values and q-values (FDR-corrected p-values) obtained by multifactorial general linear model ANOVA on factors Immulix, diet and sex, based on rarefied (10,000) OTU-tables of faecal samples from week 12. Scale on the right indicates relative abundance (%).

Beta diversity (Bray–Curtis dissimilarities) between faecal samples from week 12 was significantly altered by diet (*p* = 0.001; Fig. 5C) and Immulix (*p* = 0.010; Fig. 5D). No effect of Immulix (*p* = 0.521) or diet (*p* = 0.738) on alpha-diversity was found. When investigating differences in relative abundance at different taxonomic levels, it was found that Immulix feeding downregulated the order Lactobacillales (q = 0.035) driven by specific downregulation of the family *Streptococcaceae* (q = 0.000; Table 1and Fig. 6). While Immulix did not affect genus or species level of *Lactobacillus*, the HP diet upregulated *L. animalis* (q = 0.034). Immulix upregulated the phylum Bacteroidetes (q = 0.047), mainly driven by an increase of the family *Bacteroidaceae* (q = 0.010) and genus *Bacteroides* (q = 0.010)*.* An unspecified *Prevotella* sp. was decreased by both Immulix (q = 0.006) and the HP diet (q = 0.034). *Akkermansia* and *Bifidobacteria* were not abundant in the mice, except in four mice from the HP + Imx group, that harboured *Bifidobacterium animalis* (0.31–14.5%). No significant correlations between bacterial abundances and remaining data were identified when p-values were FDR-corrected, although some correlations between some taxa and gene expression of *Tnf*α and *Cd4* in spleen tended to be significant (*p* < 0.05; q < 0.1; Table 2).

**Table 1:**
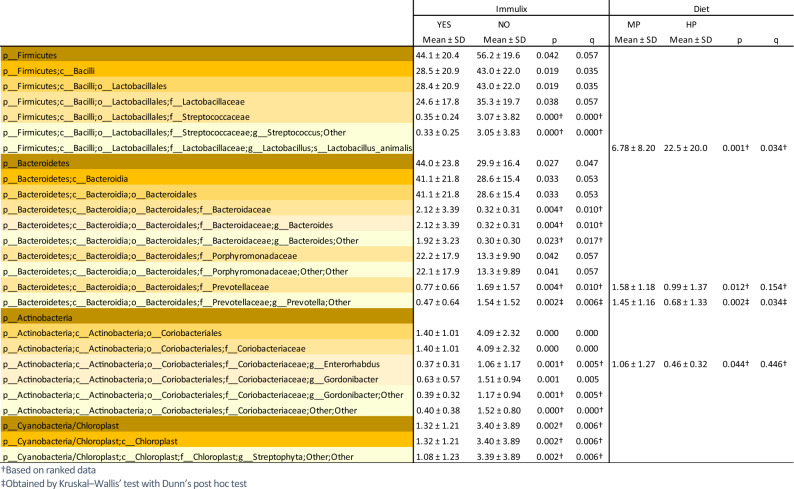
Means of relative abundance (%) with p-values and q-values (FDR-corrected p-values) obtained by multifactorial general linear model ANOVA on factors Immulix, diet and sex, based on rarefied (10,000) OTU-tables of faecal samples from week 12. Only taxa with *p* < 0.05 are listed.

**Table 2:**
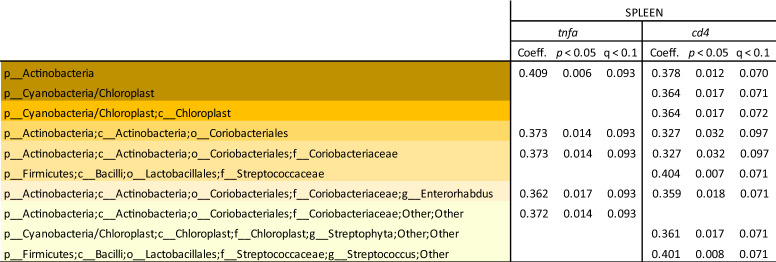
Correlation coefficients between bacterial abundances (based on rarefied (10,000) OTU-tables) and remaining data that tended to be significant (*p*-value < 0.05; q-value (FDR-corrected *p*-values) < 0.1).

### A human profile diet increased weight gain

**Figure 7:**
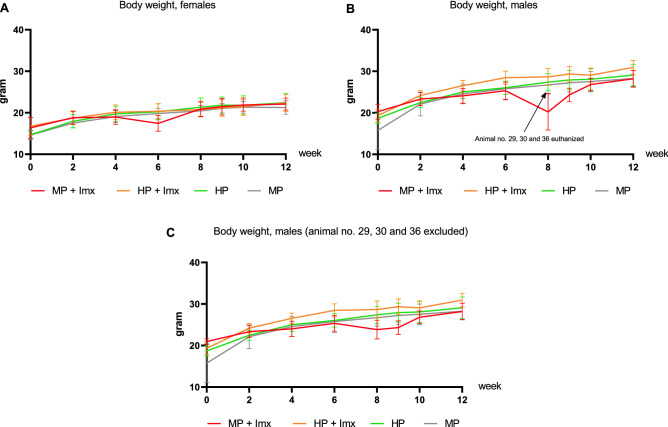
Weight gain within feeding groups over the experimental period from week 0 to week 12. Three animals from theMP + Imx group were euthanized at week 8 due to weight loss and impairment. (**A**): Body weight, females. (**B**): Body weight, males. (**C**): Body weight, males, where mouse no. 29, 30 and 36 were excluded. A multifactorial general linear model ANOVA on factors Immulix and diet revealed significantly higher weight gain (AUC) of mice fed the HP diet, compared to the MP diet (*p* = 0.034), but no significant effect of Immulix (*p* = 0.475). However, if data from mouse no. 29, 30 and 36 were excluded, this effect disappeared (Immulix *p* = 0.992; diet *p* = 0.149), indicating that the increased weight gain was caused by the abnormal weight loss from those three mice, rather than a factor-effect.

Three male mice (no. 29, 30 and 36) from the MP + Imx group from the same cage were euthanized at week 8 due to weight loss and impairment, probably caused by aggressive behaviour. There were no significant effects on body weight if these mice were not included in the data evaluation (Fig. 7).

## Discussion

The overall aim of this study was to investigate whether immune-regulating effects of Immulix influence immune responses to vaccination, as foals, which are the targets of Immulix, are routinely vaccinated. Significantly lower tetanus-specific IgG titers four weeks after secondary vaccination were observed in Immulix fed male mice compared to males not fed Immulix (*p* = 0.036). Testosterone has previously shown immunosuppressive properties on vaccine responses^45^, but van den Elsen, et al.^34^ reported increased trivalent influenza vaccine (TIV)-specific IgG titers in male mice four weeks after primary vaccination (*p* < 0.01). However, it is doubtful whether the observed difference in IgG titers between male mice fed Immulix and males not fed Immulix has any biological relevance, because they were approximately twofold, while a booster vaccination increased the response tenfold and lowered the difference. When both sexes were included in the statistical tests, no effect of Immulix was detected. In agreement with van den Elsen, et al.^34^, our study revealed significantly higher and more diverse IgG titers for females compared to males, which might explain why significant effects were only detected in males. The higher variance in females might be explained by cycle fluctuations, since oestrogens can promote B cell proliferation and Ig production^46^. In 2014, The US National Institutes of Health (NIH) introduced policies about sex inclusion, to promote equal use of males and females in cell and animal research, as present with NIH-funded clinical research^47^. However, conclusions in animal experiments are often solely based on results from either sex or results are not corrected for the sex-effect. The sex-effects observed in our study underline the importance of equally including both males and females in immunological animal experiments. The current literature supports the findings by van den Elsen, et al.^34^, that oligosaccharides have a positive effect on vaccine responses. Therefore, our findings, that oligosaccharides had a negative effect on vaccine responses in male mice, are scientifically novel. Nonetheless, it is worth to note that antibody titers are a measure of immunogenicity and do not definitely tell if the animal is fully protected against disease. Therefore, more exact conclusions about disease prevention can be obtained with challenge experiments. Since the purpose of this project have been to study the role of oligosaccharides on antibody titers as well as some ethical aspects, challenge experiments have been disemployed.

The fact that Immulix feeding upregulated *Il1b* in the spleen (*p* = 0.001), but did not affect IL-10 and T or B cell surface markers, substantiates the lack of effect on vaccine responses. An HP diet has previously proven to increase the appearance of T-cell subsets in ex-germ-free mice inoculated with a human microbiota^35^, but our study seems to indicate that in mice with their own microbiota, Immulix may dampen that effect, as Immulix fed in combination with the HP diet downregulated the expression of *Cd4* (Fig. 5A) and *Foxp3* (Fig. 5B) in ileum. As prevailing with the IgG results, sex was a considerable factor with higher expression levels of *Cd4* (*p* = 0.008) and *Cd8a* (*p* = 0.023) in ileum as well as *Cd19* (*p* = 0.005) and *Foxp3* (*p* = 0.016) in the spleen of female mice, compared to males. This was moreover the case in the study by van den Elsen, et al.^34^, in which female mice expressed significantly higher levels of IL-2, IL-6 and IL-10 in splenocyte supernatants harvested 72 h after ex vivo TIV stimulation. The T cell markers (*Cd4* and *Cd8a*) and the B cell marker (C*d19*) might be upregulated as a result of stronger vaccine responsiveness in females compared to males. Dietary fat content affected gene expression in the spleen, with higher expression of *Il1b* (*p* = 0.001), but lower levels of *Cd8a* (p = 0.035) in mice fed the HP diet, compared to MP diet fed mice. Moreno-Indias, et al.^35^ reported lower *Cd8a* expression levels in colon of ex-germ-free mice inoculated with mouse microbiota, than with human microbiota^35^, which supports that our results are specific for the outcome in mice with a murine microbiota. Also, the results by Moreno-Indias, et al.^35^ were detected in ileum and colon, indicating local responses, whereas this present study only revealed significant results in the spleen, which is more the indication of a systemic response. Interestingly, dietary fat content had an upregulating effect on *il6* and *il1b* in females (Fig. 4A and B), but a downregulating effect on *il6* and *cd4* in males (Fig. 4D and E), possibly as a result of oestrogen-driven sex differences on lipid metabolism^48^. In accordance, Wallace, et al.^49^ found reduced proinflammatory cytokines in spleen lymphocytes (either Concanavalin A stimulated or incubated with various fatty acids) from male mice fed high-fat diets (21%; coconut, safflower, or fish oil) compared to a low-fat diet (2.5%; maize oil). Unfortunately, no females were included in that experiment. As an extension to the sex differences found in our experiments, it is enviable to investigate the hormones’ role on the immune response. Oligosaccharides are known modulators of the GM and have proven to increase specific beneficial bacteria. In horses, Immulix has previously proven to increase abundances of *Akkermansia* spp.^12^, which has an immune modulating effect^50,51^. In our study, *Akkermansia* were not present in the mice, which was also the case in the characterization of faecal microbiota of BALB/c mice in the study by Krych, et al.^52^. Hänninen, et al.^51^ transferred GM from non-obese diabetic (NOD) mice with a low diabetes incidence to NOD mice with a high diabetes incidence without affecting the diabetes incidence because *Akkermansia muciniphila* was one of few taxa not successfully transferred. However, when *A. muciniphila* was orally administered, diabetes incidence decreased significantly^51^. This indicates that despite major alterations in GM, the absence of one single taxon can have considerable consequences. Especially *Lactobacilli* and *Bifidobacteria* have extensively been reported to increase as a result of oligosaccharide feeding^10,11,53,54^. In this study, *Bifidobacteria* were not found, except in four mice from the HP + Imx group, where *Bifidobacterium animalis* were abundant (0.31–14.5%), and Immulix actually had a downregulating effect on expression of *Cd4* and *Foxp3* in ileum of mice fed the HP diet. Two weeks of probiotic supplementation (containing various amounts of *Bifidobacteria* and *Lactobacilli* spp.) did not seem to be sufficient to establish colonies of *Bifidobacteria*. Several *Lactobacilli* spp*.* were abundant in this study, but unexpectedly Immulix decreased the order *Lactobacillales* (q = 0.035), whereas no significant changes were observed for *Lactobacillus animalis.* Interactions and synergistic effects of *Bifidobacteria* and *Lactobacilli* are known to occur, and these bacteria are often upregulated simultaneously^11,55,56^. Therefore, the absence of *Bifidobacteria* might have affected abundances of *Lactobacilli* as well. The genus *Bacteroides* was in this study upregulated by Immulix feeding, which might have outcompeted *Bifidobacteria*, Lactobacillales and Prevotella in those mice, which is in compliance with Hansen, et al.^57^, who found that xylooligosaccharide feeding tended to decrease *Prevotella* to the detriment of upregulated *Bacteroides* and especially *Parabacteroides*. GM composition has previously been correlated with vaccine responses. For instance, vaccine specific antibody responses have been positively correlated with *Bifidobacteria* and *Lactobacilli*^10,58^ and negatively correlated with *Clostridiales*^59^. None of those bacteria were affected by Immulix feeding or correlated with vaccine responses in our study. The Proteobacteria/Bacteroidetes ratio have furthermore proven to affect vaccine responses, with Proteobacteria being a stronger immune stimulator than Bacteroidetes^16,60^. In our study, Proteobacteria were unaffected, and *Bacteroides* spp. were upregulated in Immulix fed mice. Additionally, Immulix decreased *Prevotella*, which have been correlated with T_h_1 and T_h_17 response^61^. Hence, the differences in GM of mice fed Immulix and those not fed Immulix, do not indicate that Immulix fed mice should respond better to a vaccine. With this in mind, it is not surprising that vaccine responses, immune cells and cytokine profiles were not affected dramatically by the treatments. Therefore, it might be argued that this BALB/c murine model may not be a highly appropriate model for testing oligosaccharides, and at least this is very dependent on its gut microbiota, which varies substantially between vendors^62^. The GM of laboratory mice often has a low diversity and is very different from feral animals^63^. Therefore, when using this model for testing an equine oligosaccharide-based feed supplement, it could be considered to inoculate the mice with microbiota from foals as demonstrated by Lindenberg et al.^12^, and to orally administer specific target bacteria, which was successfully demonstrated by Hänninen et al.^51^. In order to achieve better inoculation efficiency, the mice diet could have been modulated to resemble a horse diet either with respect to macronutrient content or source, as demonstrated by Moreno-Indias, et al.^35^ for human microbiota colonization. However, it should be noted that xenografted microbiotas do not stimulate the immune response very well^64^.

In conclusion, Immulix feeding or increased dietary fat content significantly modified the GM of mice, but neither of these had any biological relevant effects on tetanus vaccine responses in the mice, despite minor influences on immune cell markers, cytokines and IgG titers. Additionally, significant sex differences existed, with female mice exhibiting higher and more diverse immune responses than males, and the antibody response of the males in contrast being negatively affected by the oligosaccharide feeding.

## Data Availability

Sequencing data is available in the Sequence Read Archive (SRA) with the accession number: PRJNA715066. Remaining raw data as well as supplementary data is available in a repository within the Open Science Framework under the name: “Oligosaccharide equine feed supplement has only minor impact on vaccine responses in mice” and can be accessed via: https://osf.io/vq6nz/.
